# The performance of retail investors, trading intensity and time in the market: evidence from an emerging stock market^☆^

**DOI:** 10.1016/j.heliyon.2021.e08583

**Published:** 2021-12-17

**Authors:** Urbi Garay, Fredy Pulga

**Affiliations:** a IESA Business School, Av. IESA, Edif. IESA, Urb. San Bernardino, Caracas, Venezuela; b International School of Economic and Administrative Sciences (EICEA), Universidad de La Sabana, Campus del Puente del Común, Km 7., Chia, Cundinamarca, Colombia

**Keywords:** Household finance, CAPM, Fama and French, Carhart, Underperformance and Colombia

## Abstract

We analyze all stock transactions executed by the universe of individual (or retail) investors of the Colombian Stock Exchange (5,380,810 trades performed by 42,211 individual investors between 2006 and 2016). Retail investors had negative abnormal returns on a *gross* excess return basis that ranged between 4% and 4.4% per year (depending on whether the alpha was estimated using the CAPM, Fama-French model or Carhart model). When transaction costs are considered, the underperformance of retail investors becomes even more pronounced, and the most active traders perform worse than less active traders even on a gross excess return basis. The underperformance of retail investors can be explained by their bad timing but only prior to the bankruptcy of Interbolsa, the largest stock brokerage house in Colombia at the time (2012). Once we control for the number of trades and other variables, we find that retail investors present in the market for a longer period of time and trading more actively outperform the other investors (on both a gross and net basis).

## 1 Introduction

Since the Global Financial Crisis of 2008-09, interest rates in many countries have declined (in many cases, to record low levels). Over the past few years, disruptive online brokers have introduced new stock (and option) trading platforms to facilitate trading by retail (individual) investors and, at the same time, have even offered zero trading commissions in recent years (Mihm, 2020). These factors, the extra time afforded to individuals globally by the COVID-19 lockdown that started in early 2020 and an increasing use of social media have brought the performance of retail investors to the forefront.^1^

On aggregate, for every investor overperforming the market, there must be another investor underperforming it, and investors must earn the market return before costs. However, we still know relatively little about the performance of households in emerging stock markets. Our aim is to shed light on the performance of individual investors in Colombia, a stock market characterized by an active investor community, relative illiquidity (except for the largest companies), relatively high transaction costs, corporate governance issues, and presumably grater information asymmetries than those found in developed markets (Wyman, 2016).

We study the performance of retail investors in Colombia making use of a unique dataset of transactions corresponding to 5,380,810 trades executed on behalf of 42,211 individual investors during the 2006–2016 period. The database covers the complete trading history of all retail investors in Colombia for this period. This is an advantage of our study relative to other research only using a sample of traders (for example, traders belonging to a certain brokerage house) to examine the performance of individual investors. Our study also analyzes the performance of individual investors according to the intensity of their trading activity and examines whether timing, as well as experience (proxied by the length of time an investor has been in the market), can explain the performance of individual investors. The paper also allows us to test the predictions of the rational expectations model proposed by Grossman and Stiglitz (1980) and to compare them to those of a model in which investors suffer from overconfidence bias (Odean, 1999). For Daniel and Hirshleifer (2015, p. 1), “…Overconfidence means having mistaken valuations and believing in them too strongly.”

The data that we use have the following positive features. The data include all transactions executed in a specific stock market (the Colombian Stock Exchange), comprise a relatively long period of time (between January 2, 2006 and January 29, 2016), and allow us to identify all transactions made by each individual investor in the database. Pedraza et al. (2019) have already used this database to analyze the trading behavior and performance of foreign investors with different management styles (we comment more on this paper below). However, whereas their paper focuses on the trading behavior and performance of foreign institutional investors relative to domestic institutional investors, our paper concentrates on the trading behavior and performance of individual investors on an aggregate level in relation to the intensity of their trading and on the impact of trading characteristics on their performance.

We find that households had negative abnormal returns on a *gross* excess return basis that ranged from 4% to 4.4% per year (alphas vary slightly depending on whether they were estimated using the CAPM, Fama-French model or Carhart model). When transaction costs are considered, the underperformance of retail investors becomes even more dismal. The average individual investor biases its portfolio toward small, low-beta stocks and turns over approximately 8.8% of its portfolio monthly. Additionally, the most active traders perform worse than less active traders even on a *gross* excess return basis. When we divide the sample period into two subperiods, we find that the alpha was approximately 2% more negative in the first subperiod (01-2006/10-2012) than in the second subperiod (11-2012/01-2016). We also find that individual investors had bad timing over the whole sample period, explaining their inferior performance, but this bad timing disappeared after the bankruptcy of Interbolsa (which occurred in October of 2012), the largest stock brokerage firm in Colombia, suggesting that the worst underperformers might have disappeared after this event. Finally, we find that investors present in the market for a longer period of time (a variable that can be regarded as a proxy for experience) and trading more actively outperform the other investors (on both a gross and net basis). To the best of our knowledge, this paper is the first to offer evidence of the positive effect that the combination of trading intensity and experience may have on investor returns.

Section 1 presents a literature review, where rational expectations and behavioral finance models on the expected performance of individual investors are discussed, and their associated empirical evidence is presented. Section 2 examines the data and presents the methodology followed in the study; Section 3 presents the results of the paper, including a number of robustness checks and extensions; and Section 4 offers conclusions, potential extensions and implications of the paper.

## 2 Literature review

The study of the trading behavior and performance of individual investors has gained increasing interest over the past two decades. In research conducted up to the late 1990s, the study of investor performance concentrated mostly on the performance of institutional investors and, more specifically, on the performance of equity mutual funds. At the time, data on mutual fund returns were readily available, and economists tested whether investors were unable to earn superior risk-adjusted returns after reasonably accounting for transaction and opportunity costs (Barber and Odean, 2013). On the institutional side of the market, most studies of mutual funds conclude that the performance of the average fund lags behind that of the aggregate market and that only little evidence exists in support the proposition that the few mutual funds that do outperform exhibit persistence. This issue is, however, far from having been settled.

Barber and Odean (2000) conducted a seminal study in which they analyzed the position statements and trading activity of 78,000 households under a large discount brokerage firm between 1991 and 1996, finding evidence suggesting that overconfidence leads to excessive trading. While Barber and Odean (2000) found that there is very little difference between the gross performance of households that trade frequently and those that trade infrequently and found a significant difference in returns when transaction costs (e.g., commissions, market impact, bid-ask spreads, and transaction taxes) are taken into account. More specifically, whereas households that trade frequently earn a net mean annual return of 11.4%, investors that trade infrequently earn 18.5%. These results are consistent with models in which trading originates from investor overconfidence and are inconsistent with models according to which trading arises from rational expectations. Barber and Odean (2000) also found that after accounting for transaction costs, individual investors significantly underperform on relevant benchmarks. The authors also conclude that households trade common stocks frequently, trading costs are high (approximately 3% in commissions and 1% in bid-ask spread), and households tilt their investments toward small high-beta stocks.

Barber and Odean (2013) provide a synopsis of research on individual investors and their stock trading behavior and document that the behavior of such investors detrimentally affects their financial wellbeing because they underperform on standard benchmarks, suffer from the “disposition effect” (they tend to sell winning investments while keeping their losing investments), are heavily influenced by past return performance and limited attention in their purchase decisions, avoid past behaviors that have generated pain while repeating past behaviors that have coincided with pleasure, and tend to hold undiversified stock portfolios.^2^ The disposition effect is often cited as one of the main determinants of the performance recorded by individual investors.

Previously, Grossman and Stiglitz (1980) developed a rational expectations model in which a group of investors choose to invest passively and others choose to acquire costly information. While in equilibrium all investors have the same expected utility, active and informed investors earn higher precost returns. These higher precost returns are needed to compensate investors for the expenses associated with information gathering and processing and are no longer abnormal when they are accurately accounted for. In a large liquid market, information costs probably explain only small abnormal returns.

According to Lee et al. (1991), investor sentiment is normally attributed to individual retail investors. Individual investors tend to place small trades, and their purchases and sales must be correlated if they are to substantially influence prices (Barber et al., 2009). The authors use transaction data and identify buyer- or seller-initiated trades to study the trading of individual investors. The authors find that individual investors herd, that small trade order imbalances forecast future returns, that small trade order imbalances are correlated with order imbalances based on trades (from retail brokers), and that, over a weekly horizon, small trade order imbalances reliably predict returns.

Barber et al. (2009) analyze the performance of individual investors in Taiwan (the world's 12th largest stock market at the time of the study) using a unique and complete dataset including complete transaction data, the identity of each trader and underlying order data for the period of 1995–1999. The authors find that trading in financial markets by individual investors causes economically large losses and that nearly all of the losses can be traced to their aggressive orders. The authors also construct portfolios that mimic the buying and selling of each investor and find that while individuals lose, each group of institutional investors analyzed (corporations, foreigners, dealers, and mutual funds) wins. Furthermore, most of the losses by individuals and gains by institutions occur within a few weeks of trading.

In another important country-based study, Linnainmaa (2010) uses data from the Finnish Stock Exchange for 1998 to 2001 to identify whether an investor has placed a market or a limit order, a feature that was made possible by the characteristics of the Finish dataset. Linnainmaa (2010) finds that individual investors underperform related to informed traders because the latter select their limit orders. More specifically, the returns on individual investor trades that originate from limit orders lose 0.51% on the day following a trade and 3.3% over a 63-day period. However, whereas individual investors in Finland lose money on executed limit orders, they make money on executed market orders. When gains and losses are combined, the profits are not significantly different from zero. However, the evidence from Taiwan, which has an electronic limit order market, differs from the evidence from Finland.

Most existing empirical research suggests that individual investors behave differently from investors in rational expectations models. For example, many apparently uninformed investors trade speculatively, actively, and to their detriment, and most retail investors hold undiversified portfolios. Furthermore, when viewed as a group, individual investors make systematic (not random) buying and selling decisions. A salient finding in the literature is that most research concludes that the average long-run performance of individual investors is poor even before costs. However, and contrary to the long-run evidence, there is evidence that indicates that the returns obtained by individual investors over very short horizons (up to a week) seem to be fairly strong. For example, Kaniel et al. (2008) find that the stocks bought by individuals in the 10 days preceding an earnings announcement outperform, on average, those sold in the two days around the earnings announcement by approximately 1.5%.

There exists great variation in performance across individual investors, and this cross-sectional variation in performance can be traced to cognitive abilities (for example, Korniotis and Kumar, 2009a, conclude that smarter investors outperform others by approximately 3.6% per year), IQ (Grinblatt et al., 2012, determine that in Finland, the spread in returns earned by low versus high IQ investors is a significant 2.2% per year), experience (Campbell et al., 2014, document that both investment experience and feedback from investment returns affect investor behavior, performance, and favored stock styles), age (Korniotis and Kumar, 2009b, find that investment performance declines with age), optimism (Kaplanski et al., 2015, find that individuals with more positive sentiment also have higher return expectations and a higher intention to buy stocks), and gender (Barber and Odean, 2001, find that men perform worse than women and relate these results to the fact that men trade more often than women because they are more prone to overconfidence). The cross-sectional variation in performance has also been linked to investment skill, investment style, and location.

A few notable studies find evidence that individual investors overperform in the market. For example, Ivkovic et al. (2008) find evidence that individual investors with relatively concentrated portfolios outperform individuals who are more diversified. Ivkovic and Weisbenner (2005) show that individuals outperform the market when they buy the stocks of companies that operate close to their homes (compared to the stocks of far-away companies). Finally, and as noted above, Kaniel et al. (2008) find that stocks heavily purchased by individuals in one month generate positive excess returns in the subsequent month. A criticism of the previous findings is that given the low diversification of many of the accounts of these studies, one would expect substantial cross-sectional variation in performance by chance (even if there are no differences in skill; see Barber and Odean, 2013).

Sicherman et al. (2015) find that daily investor online account logins fall by 9.5% after the market declines and that investors also pay less attention to the market when volatility (measured using the VIX volatility index) is high. The level of attention and the correlation between attention and returns are strongly related to financial position (wealth and holdings) and to investor demographics (age and gender). Andries and Haddad (2019) developed a life-cycle model in which underdiversification can be generated by preference-based utility costs of information because investors choose to have only a few stocks in their portfolios to reduce the likelihood of receiving disappointing information. In a recent paper, Quispe-Torreblanca et al. (2020) use data on brokerage account logins by individual investors and find that individuals devote disproportionate attention to already-known positive information about the performance of individual stocks that they have in their portfolios and increase trading after recent gains. Conversely, these individuals display an aversion to paying attention to unfavorable information, which reduces trading after recent losses. These findings are consistent with individual investors exhibiting “information aversion” (Andries and Haddad, 2019). Previously, Olafsson and Pagel (2017) found that attention to financial accounts by individuals is increasing for cash holdings, savings, and liquidity and decreasing for spending and overdrafts. Finally, Song (2020) finds that even though attention to accounting information by individual investors is high, their acquisition of accounting information does not necessarily contribute to informationally efficient capital markets.

We still know little about the performance of individual investors in emerging markets (with the exception of that in Taiwan, which is nonetheless a relatively large market among the emerging markets and one where day trading is very prominent). However, Campbell et al. (2014) use data on Indian stock portfolios and find that both years of investment experience and feedback from investment returns have significant effects on investor behavior, performance and favored stock styles. In a subsequent and more recent paper, Campbell et al. (2019) find return heterogeneity to be the most important contributor explaining the increasing inequality of wealth held in risky assets by Indian individual investors.

The following are further questions that we attempt to answer in this paper. Are retail investors able to time the market? Do trading experience and trading intensity affect the returns of retail investors? How does the performance of institutional investors with regard to trading intensity compare to that of retail investors?

## 3 Data and methodology

The dataset that we use was provided by the Colombian Stock Exchange and includes all stock transactions executed between January 2, 2006 and January 29, 2016.^3^ For each transaction, the dataset includes the date and time of the transaction, the transaction price, the number of shares traded, the name of the company, whether the order was a buy or sell trade, a stock identifier, and a broker ID. As noted by Pedraza et al. (2019), who used these data to compare the performance of domestic versus foreign institutional investors in Colombia, a key aspect of the dataset, and one that is very relevant to us, is that every purchase and sell record includes a distinctive investor ID number that allows for the identification of each transaction executed on behalf of each investor throughout the sample period. It is important to highlight here that most of the existing empirical literature proxies the transactions performed by individual investors as those that correspond to small trades. However, we have the advantage of being able to identify whether a transaction corresponds to an individual or to an institutional investor because we have access to a distinct investor ID. As the main limitations of the database, we do not have information on the demographic characteristics of retail investors and nor a record of the order book for stocks.

The Colombian stock market had a market capitalization of USD 11.1 billion at the beginning of the study period (2006). The highest market capitalization level of the sample period was reached in 2010 (USD 15.5 billion), and it later declined to USD 8.6 billion in December of 2005.

The initial dataset includes all buy and sell stock trades and excludes repurchase agreements and shares tendered for short sales.^4^^,^^5^ We also exclude international stocks, domestic stocks for which there were less than one thousand transactions during the sample period, and investors that transacted less than 20 times during the whole sample period. On average, such traders executed only two trades during the sample period, and thus, we categorize them as “buy and hold” investors with no meaningful trading activity. We also discard transactions of below 154,500 Colombian Pesos (equivalent to between approximately USD 50 and USD 75 depending on the exchange rate of the respective date). We also exclude investors who did not perform trades for periods of at least 24 months.

Stocks that did not trade for three or more months were also excluded. We excluded “special issues” of non-dividend common stocks, which represent 14% of the total securities included in our database. Our main concern with these securities is that there was actually no trading activity on them. Such stocks are traded mostly by “buy and hold” investors.

The application of the previous filters led to the exclusion of 70% of the stocks and 92% of the accounts present in the database. The exclusion of stocks with low trading activity and “buy and hold” investors allows us to keep 77% of the trades. To analyze the results by trading activity, we divide the data by quantiles of trades.^6^ Data show that the top quantile of trades encompasses 80% of the transactions; therefore, the results of the regressions of aggregate investors are mainly explained by this group. This result implies that adding “buy and hold” investors to the dataset should not divert our main results. We are left with 42,211 individual investors and 5,380,810 transactions. Stock prices were adjusted for splits and other corporate events that affected their nominal value.

We also calculate the variable “spread,” measured as in Barber and Odean (2000). This is a relative measure of the buy or sell price against the daily closing price. We found that the mean value of this variable reached only -0.09%, suggesting that the stocks in our final database are relatively liquid stocks.

We do not know the exact portfolio that each investor has in the dataset. Therefore, and similar to Pedraza et al. (2019), we created investor portfolios assuming a zero initial holding condition for each investor for the first date that he or she appears in the dataset. We then added daily stock holdings to each investor over time. For each investor, we identified negative holdings at the end of the whole period of the dataset and added this value to the zero initial holdings to account for the short-sell ban. Errors in our measure from differences in initial conditions should decline over time if portfolio turnover is large enough and considering that the sample period spans 10 years.

Each individual investor's portfolio return is calculated monthly as (P_t_ – P_t-1_)/P_t-1_ where P_t_ is the value of the portfolio at the end of the month and P_t-1_ is the value of the portfolio at the beginning of the month. P_t_ is found by multiplying the number of stocks of each company in the investor's portfolio by its closing price at *t* and summing them. The value of the portfolio includes cash dividend payments. We obtained stock returns, share prices, trading volumes, and bid-ask spreads from Bloomberg, Datastream and the Colombian Stock Exchange.

We measure investor turnover as the maximum between the sum of monthly buys and sells by an investor divided by the value of the portfolio.

We calculate three measures of risk-adjusted performance. First, we calculate the alpha starting with the CAPM (Jensen's alpha). We estimate the following monthly time-series regression in which we regress the monthly excess return earned by each individual investor (*r - r*_*f*_) on the market excess return (Eq. (1)):

(1) $$r-r_{f}=\alpha +\beta_{1}\left(r_{m}-r_{f}\right)+\varepsilon$$

where *r*_*f*_ is the risk-free rate and (*r*_*m*_ *- r*_*f*_) is the market excess return.

Second, we employ an intercept test using the three-factor model developed by Fama and French (1993), also known as the three-factor alpha. Thus, to evaluate the performance of individuals in aggregate, we estimate the following monthly time-series regression (Eq. (2)):

(2) $$r-r_{f}=\alpha +\beta_{1}\left(r_{m}-r_{f}\right)+\beta_{2}\left(SMB\right)+\beta_{3}\left(HML\right)+\varepsilon$$

where *r*_*f*_ is the risk-free rate, (*r*_*m*_ *- r*_*f*_) is the market excess return, *SMB* is calculated as the return on a value-weighted portfolio of small stocks minus the return on a value-weighted portfolio of large stocks, and *HML* is calculated as the return on a value-weighted portfolio of high book-to-market stocks minus the return on a value-weighted portfolio of low book-to-market stocks. Barber and Odean (2000) argue that one should place particular attention on the Fama–French intercept tests, as individual investors tend to bias their portfolios toward small stocks and the three-factor model offers a reasonable adjustment for this small stock bias.

The third intercept test is performed using the Carhart four-factor model (Eq. (3)). This intercept is known as the four-factor alpha. The Carhart four-factor model adds a momentum factor to the three-factor model of Fama and French. Momentum (*WML*) is the tendency for stock prices to continue to rise when a stock price is increasing and to continue to decrease when stock prices are declining. Momentum is calculated by constructing a zero-cost portfolio that is long for the previous 12-month return winner stocks and short for the previous 12-month loser stocks, reflecting the monthly premium on winners minus losers.

(3) $$\left(r-r_{f}\right)=\alpha +\beta_{1}\left(r_{m}-r_{f}\right)+\beta_{2}\left(SMB\right)+\beta_{3}\left(HML\right)+\beta_{4}\left(WML\right)+\varepsilon$$

The portfolios for the factors (i.e., market, *SMB*, *HML* and *WML*) for Colombia were calculated by Pedraza et al. (2019), who kindly provided these data to us. The risk-free rate that we employed is the one-month Colombian T-bill rate.

## 4 Results

During the sample period of 2006–2016, aggregate stock market capitalization in Colombia ranged from a minimum of 34% of GDP in 2005 to a maximum of 73% in 2012 (ending at 35% in 2015), and the number of listed firms started at 68 in 2005 and ended at 65 firms in 2015. According to evidence presented by Pedraza et al. (2019), between 2006 and 2010, domestic individual investors accounted for 46% of all stock transactions (in total value) made in the Colombian Stock Exchange and for 27% between 2011 and 2016.^7^ The decline between the two subperiods could be attributed to the effects of the Global Financial Crisis of 2008-09 on subsequent years, which might have affected individual investors more harshly than institutional investors, and the negative effects of the bankruptcy of Interbolsa, the largest stock brokerage in Colombia, in late 2012. This brokerage firm had close to 20,000 clients, and its demise was a shock that affected the confidence of investors in the system.

Table 1 shows the descriptive statistics of our database. Panel A shows that a total of 5,380,810 trades were executed on behalf of 42,211 individuals. The mean number of trades reached 127.47 and the median reached 44. The number of trades is strongly skewed to the right, similar to volume and trade size (both of which are measured in Colombian pesos or COP). This skewed-to-the-right behavior has also been documented by Barber and Odean (2000) for the U.S. and by Barber et al. (2009) for Taiwan, among other papers, wherein a small %age of all households are responsible for a large fraction of all trades and volume executed. Mean commissions reached 2.36% (median commissions reached 2.07%). The mean commission declines as the volume increases, as the commissions charged by brokerage houses decrease when the total volume traded by the investor increases. The monthly mean turnover reached 8.82, a number that is slightly higher than the figure reported by Barber and Odean (2000) of approximately 6.0.

**Table 1:** Descriptive statistics. The sample of individual investors at the Colombian Stock Exchange from 2006 to 2016 records 5,380,810 trades of 42,211 individuals. Panel A exhibits descriptive statistics calculated per transactions: Trades is the number of transactions, either buys or sells, during the period performed by each investor. Volume is the monetary amount traded by investor, expressed in Million COP (Colombian Pesos). Trade size is expressed in Million COP. Commissions are calculated as the total commission expense divided by the total volume traded by investor, and is expressed in percentage. Monthly turnover is calculated as the maximum of monthly buys or sells divided by the value of shares held during the month. Panel B exhibits descriptive statistics of monthly raw returns of individual investors expressed in percentages (i.e. not risk-adjusted): Raw is the return of the portfolio for every investor, calculated as the ratio of the market value of her portfolio at the end and the beginning of each month minus one, including dividends. Net is calculated as the ratio of the market value of her portfolio at the end and the beginning of each month minus one, including dividends, net of commissions.

	Mean	25th Percentile	Median	75th percentile	Sd	Observations
Panel A. Trades descriptive statistics						
Trades	127.47	27	44	89	648.68	5,380,810
Volume	4,547	341	758	2,036	41,205	5,380,810
Trade size	35.67	12.63	17.23	22.88	63.52	5,380,810
Commissions	2.36	1.76	2.07	2.70	1.04	5,380,810
Monthly turnover	8.82	6.53	8.30	10.23	4.11	5,380,810

Panel B. Individuals raw returns descriptive statistics						

Raw	-0.0404	-0.1185	-0.0309	0.0541	0.2167	42,211
Net	-0.0632	-0.1379	-0.0487	0.0330	0.2312	42,211

Panel B of Table 1 shows the raw returns for the sample of 42,211 individual traders for between 2006 and 2016. It is worth noting that the mean gross monthly returns were negative (−0.04%) with a standard deviation of 0.22%. Mean net monthly returns were lower (−0.06%) and had a standard deviation of 0.23%. Both gross and net returns become monotonically more negative from the first to fifth quintiles of trading intensity (these results are not reported in the table). It is important to note that whereas the gross and net raw returns of individual investors reported in Barber and Odean (2000) were positive for all quintiles, for Colombia, all of the raw returns of individual investors (both gross and net and for all quintiles) are negative.

The results of the panel regressions on the three measures of risk-adjusted performance (the CAPM, Fama and French model or 3-factor model and the Carhart or 4-factor model) are presented in Table 2. Whereas Panel A of the table presents the results for the gross return performance of aggregate individual investors, Panel B shows the net return results. The results of the three models clearly indicate that households underperform even in terms of gross returns (recall that Barber and Odean, 2000, found very little difference between the gross performance of individual investors that trade frequently and those that trade infrequently). The alphas range from -0.34% per month to -0.37% per month on a gross return basis, and underperformance is even worse when transaction costs are included. The six alphas are negative and statistically significant at 1%. Underperformance by individual investors represents between 4% and 4.4% on an annualized basis. These results strongly suggest that individual investors in Colombia would have earned higher returns if they had invested in the aggregate stock market.

**Table 2:** Individual investors’ performance. This table reports estimates of the CAPM, the Fama and French 3-factor model and the Carhart 4-factor model for individual investors at the Colombian Stock Exchange from 2006 to 2016. The dependent variables are the gross and net returns in excess of the one-month Colombian T-bill rate. Market is the return in excess of the Colombian market index (COLCAP) relative to the one-month T-bill rate. SMB is the return on a portfolio of small cap stocks minus big cap stocks. HML is the return on a portfolio long in high book-to-market ratio and short in low book-to-market ratio. WML is the return on a portfolio long on winner stocks during the previous 12 months and short on loser shares during the same period. Note: t-statistics in parentheses. ∗/∗∗/∗∗∗ indicate that the coefficient estimates are significantly different from zero at the 10%, 5%, 1%, respectively.

	Gross risk-adjusted return			Net risk-adjusted return		
	CAPM	3-Factor	4-Factor	CAPM	3-Factor	4-Factor
Market	0.356∗∗∗	0.385∗∗∗	0.392∗∗∗	0.356∗∗∗	0.384∗∗∗	0.391∗∗∗
	(17.46)	(17.33)	(17.42)	(17.29)	(17.12)	(17.21)
SMB		0.120∗∗∗	0.132∗∗∗		0.118∗∗∗	0.130∗∗∗
		(2.93)	(3.20)		(2.86)	(3.12)
HML		-0.0426	-0.0434		-0.0402	-0.0410
		(-1.10)	(-1.13)		(-1.03)	(-1.05)
WML			0.0579			0.0581
			(1.60)			(1.59)
Constant	-0.00371∗∗∗	-0.00343∗∗∗	-0.00355∗∗∗	-0.00392∗∗∗	-0.00364∗∗∗	-0.00376∗∗∗
	(-3.11)	(-2.94)	(-3.06)	(-3.25)	(-3.09)	(-3.21)
Observations	119	119	119	119	119	119
R-squared	0.7227	0.7425	0.7481	0.7187	0.7379	0.7436

We now turn to our analysis of the coefficient estimates on the market, size, book-to-market, and momentum factors. On aggregate, and as expected, individual investors tend to bias their portfolios toward small stocks, as suggested by the positive and small coefficients on *SMB* for both the three- and four-factor models (the results are significant at the 1% level). Furthermore, the market beta for stocks held by households is less than one, which is intriguing and suggests that individual investors hold a portfolio of stocks that differs in some respects from the market portfolio. Individual investors in Colombia have slightly negative exposure to the *HML* factor and show a slight tilt toward momentum (*WML*), with both coefficients significant at the 1% level in all specifications. We can compare these results to those presented by Pedraza et al. (2019) for institutional investors in Colombia. The exposure of these investors (both domestic and foreign) to factors of the CAPM and Carhart model (the two models that they use) is, as opposed to our case, higher, although still lower than one (in terms of exposure to the market factor). Institutional investors exhibit limited and negative exposure to the *SMB* factor, which is consistent with institutional investors tilting their portfolios toward larger companies.

### 4.1 Performance and trading

In the rational expectations model proposed by Grossman and Stiglitz (1980), investors will trade until the marginal benefit of transacting equates to or exceeds the marginal costs of the trade, including information costs. According to this model, active and passive investors have the same expected utility. On the other hand, in models in which investors suffer from overconfidence, investors will trade excessively, and trading, at the margin, will decrease their expected utility (see, for example, the model proposed by Odean, 1999). Overconfidence bias is suffered by investors that are absolutely confident of their decisions, leading to overestimations or exaggerations (for a review of overconfidence bias, see Daniel and Hirshleifer, 2015).

As explained by Barber and Odean (2000), in the model developed by Grossman and Stiglitz, active traders must earn higher expected gross returns to compensate for their higher trading costs. Furthermore, this model predicts that the gross risk-adjusted return performance of the most active investors will be higher than that of investors with low turnover, but both types of investors will have similar net risk-adjusted returns. On the other hand, the overconfidence model predicts that the net return performance of investors with high turnover will be lower than that of investors with low turnover while making no prediction about the differences in gross returns.

To test the predictions of these two competing models with regard to trading and performance, we follow a procedure similar to that employed by Barber and Odean (2000) by dividing the sample of investors into quintiles based on total trades. We then proceed to estimate the parameters of the CAPM, Fama and French's three-factor model, and Carhart's four-factor model for each quintile by trade. The results are shown in Table 3 (once again, whereas Panel A of the table presents the results for the gross return performance of individual investors in aggregate, Panel B shows the net return results). First and importantly, the gross return performance, which is negative for each quintile, deteriorates monotonically as one moves from the lowest to the highest quintile by trading, regardless of the model used to measure performance. Whereas low trading intensity quintiles underperform by between 0.29% and 0.32% per month, high trading intensity quintiles do so by between 0.40% and 0.43%. The difference in gross return performance between investors in the highest quintile by trade and the lowest ranges from 1.3% to 1.5% (on an annualized basis) depending on the model used. These results are clearly at odds with the prediction of the model of Grossman and Stiglitz (1980) and can be accommodated to the model proposed by Odean (1999). In short, households that traded the most were not only unable to earn higher gross returns but also underperformed related to investors who traded less on a gross return basis. Furthermore, the coefficient estimates reveal that investors with higher trading activity tilt their portfolios more heavily toward low-beta and momentum stocks and less heavily toward value stocks than households that engage in less trading.

**Table 3:** Individual investors’ performance by trades. This table reports estimates of the CAPM, the Fama and French 3-factor model and the Carhart 4-factor model for individual investors at the Colombian Stock Exchange from 2006 to 2016 sorted by quantiles of trades. Panel A exhibits the coefficient estimates on gross excess returns and Panel B on net excess returns. Market is the return in excess of the Colombian market index (COLCAP) relative to the one-month T-bill rate. SMB is the return on a portfolio of small cap stocks minus big cap stocks. HML is the return on a portfolio long in high book-to-market ratio stocks and short in low book-to-market ratio stocks. WML is the return on a portfolio long on winner stocks during the previous 12 months and short on loser shares during the same period. Note: t-statistics in parentheses. ∗/∗∗/∗∗∗ indicate that the coefficient estimates are significantly different from zero at the 10%, 5%, 1%, respectively.

Trades	CAPM		3-Factor				4-Factor				
	Alpha	Market	Alpha	Market	SMB	HML	Alpha	Market	SMB	HML	WML
Panel A. Gross risk-adjusted returns											
Group 1 - Low	-0.00320∗∗	0.402∗∗∗	-0.00289∗∗	0.431∗∗∗	0.127∗∗∗	-0.0559	-0.00300∗∗	0.437∗∗∗	0.138∗∗∗	-0.0566	0.0530
	(-2.46)	(18.14)	(-2.28)	(17.81)	(2.85)	(-1.33)	(-2.37)	(17.78)	(3.06)	(-1.35)	(1.34)
Group 2	-0.00342∗∗∗	0.385∗∗∗	-0.00313∗∗	0.413∗∗∗	0.121∗∗∗	-0.0527	-0.00324∗∗∗	0.419∗∗∗	0.132∗∗∗	-0.0534	0.0513
	(-2.75)	(18.12)	(-2.58)	(17.80)	(2.84)	(-1.30)	(-2.67)	(17.77)	(3.05)	(-1.32)	(1.35)
Group 3	-0.00368∗∗∗	0.358∗∗∗	-0.00338∗∗∗	0.388∗∗∗	0.124∗∗∗	-0.0434	-0.00350∗∗∗	0.395∗∗∗	0.135∗∗∗	-0.0441	0.0556
	(-3.02)	(17.21)	(-2.85)	(17.13)	(2.97)	(-1.10)	(-2.96)	(17.18)	(3.22)	(-1.12)	(1.50)
Group 4	-0.00388∗∗∗	0.341∗∗∗	-0.00360∗∗∗	0.369∗∗∗	0.116∗∗∗	-0.0406	-0.00372∗∗∗	0.376∗∗∗	0.128∗∗∗	-0.0414	0.0570
	(-3.35)	(17.25)	(-3.19)	(17.13)	(2.92)	(-1.08)	(-3.31)	(17.23)	(3.19)	(-1.11)	(1.62)
Group 5 - High	-0.00429∗∗∗	0.299∗∗∗	-0.00402∗∗∗	0.329∗∗∗	0.116∗∗∗	-0.0254	-0.00417∗∗∗	0.338∗∗∗	0.131∗∗∗	-0.0264	0.0712∗∗
	(-3.82)	(15.61)	(-3.69)	(15.83)	(3.03)	(-0.70)	(-3.88)	(16.17)	(3.41)	(-0.74)	(2.12)

Panel B. Net risk-adjusted returns											
Group 1 - Low	-0.00347∗∗∗	0.401∗∗∗	-0.00317∗∗	0.430∗∗∗	0.124∗∗∗	-0.0533	-0.00328∗∗	0.436∗∗∗	0.135∗∗∗	-0.0540	0.0529
	(-2.65)	(17.95)	(-2.47)	(17.58)	(2.77)	(-1.25)	(-2.56)	(17.55)	(2.97)	(-1.27)	(1.32)
Group 2	-0.00366∗∗∗	0.384∗∗∗	-0.00337∗∗∗	0.412∗∗∗	0.119∗∗∗	-0.0500	-0.00349∗∗∗	0.418∗∗∗	0.130∗∗∗	-0.0507	0.0518
	(-2.92)	(17.94)	(-2.74)	(17.58)	(2.76)	(-1.22)	(-2.84)	(17.56)	(2.97)	(-1.24)	(1.35)
Group 3	-0.00388∗∗∗	0.358∗∗∗	-0.00359∗∗∗	0.387∗∗∗	0.122∗∗∗	-0.0411	-0.00371∗∗∗	0.394∗∗∗	0.134∗∗∗	-0.0418	0.0556
	(-3.16)	(17.03)	(-2.99)	(16.92)	(2.90)	(-1.03)	(-3.10)	(16.96)	(3.13)	(-1.05)	(1.49)
Group 4	-0.00406∗∗∗	0.341∗∗∗	-0.00379∗∗∗	0.369∗∗∗	0.114∗∗∗	-0.0381	-0.00391∗∗∗	0.376∗∗∗	0.126∗∗∗	-0.0388	0.0571
	(-3.47)	(17.09)	(-3.32)	(16.93)	(2.85)	(-1.00)	(-3.44)	(17.03)	(3.11)	(-1.03)	(1.61)
Group 5 - High	-0.00443∗∗∗	0.299∗∗∗	-0.00416∗∗∗	0.329∗∗∗	0.114∗∗∗	-0.0231	-0.00432∗∗∗	0.338∗∗∗	0.129∗∗∗	-0.0241	0.0719∗∗
	(-3.92)	(15.49)	(-3.79)	(15.69)	(2.97)	(-0.63)	(-3.98)	(16.03)	(3.35)	(-0.67)	(2.12)

### 4.2 Robustness checks and extensions

As a first robustness check of our main results, we use volume traded (expressed in Colombian pesos) as an alternative measure of trading activity (instead of the number of transactions previously used), and we also divide the sample by quintile based on trading commissions spent by investors. The results are presented in Table 4 (for ease of presentation, we show only the results for the alpha for each model and quintile).^8^ Panel A presents the results of the CAPM, Panel B presents the results of the Fama and French 3-factor model, and Panel C presents the results of the Carhart 4-factor model. For each panel, we first again present the alphas shown in Table 3 (trades), followed by the alphas obtained when quintiles are determined by the volume traded and then by the alphas that correspond to the quintiles classified by trading commissions. In the case of volume, and similar to the case of the number of trades used as the base case before, the alpha is negative for each quintile, and it declines as one moves from the lowest to the highest quintiles by volume on both a gross and net return basis and regardless of the model used. A similar result is found when we construct the quintiles based on trading commissions spent. These results reinforce the message that higher levels of trading activity are associated with inferior performance, even on a gross return basis.

**Table 4:** Abnormal returns. This table reports estimates of the abnormal returns of the CAPM, the Fama and French 3-factor model and the Carhart 4-factor model for individual investors at the Colombian Stock Exchange from 2006 to 2016 sorted by quantiles of trades, volume and commissions (%). Panel A exhibits the estimates on the CAPM model, Panel B on the Fama and French 3-Factor model, and Panel C on the Carhart 4-Factor model. Note: t-statistics in parentheses. ∗/∗∗/∗∗∗ indicate that the coefficient estimates are significantly different from zero at the 10%, 5%, 1%.

	Gross risk-adjusted return					Net risk-adjusted return				
	1	2	3	4	5	1	2	3	4	5
Panel A. CAPM										
Trades	-0.00320∗∗	-0.00342∗∗∗	-0.00368∗∗∗	-0.00388∗∗∗	-0.00429∗∗∗	-0.00347∗∗∗	-0.00366∗∗∗	-0.00388∗∗∗	-0.00406∗∗∗	-0.00443∗∗∗
	(-2.46)	(-2.75)	(-3.02)	(-3.35)	(-3.82)	(-2.65)	(-2.92)	(-3.16)	(-3.47)	(-3.92)
Volume	-0.00354∗∗∗	-0.00352∗∗∗	-0.00360∗∗∗	-0.00370∗∗∗	-0.00413∗∗∗	-0.00388∗∗∗	-0.00375∗∗∗	-0.00379∗∗∗	-0.00386∗∗∗	-0.00425∗∗∗
	(-2.87)	(-2.89)	(-2.96)	(-3.06)	(-3.61)	(-3.10)	(-3.05)	(-3.09)	(-3.18)	(-3.69)
Commissions	-0.00358∗∗∗ (-2.80)	-0.00368∗∗∗ (-3.10)	-0.00377∗∗∗ (-3.27)	-0.00378∗∗∗ (-3.25)	-0.00374∗∗∗ (-3.09)	-0.00372∗∗∗ (-2.89)	-0.00384∗∗∗ (-3.20)	-0.00394∗∗∗ (-3.39)	-0.00400∗∗∗ (-3.41)	-0.00410∗∗∗ (-3.33)

Panel B. 3-Factor Model										
Trades	-0.00289∗∗	-0.00313∗∗	-0.00338∗∗∗	-0.00360∗∗∗	-0.00402∗∗∗	-0.00317∗∗	-0.00337∗∗∗	-0.00359∗∗∗	-0.00379∗∗∗	-0.00416∗∗∗
	(-2.28)	(-2.58)	(-2.85)	(-3.19)	(-3.69)	(-2.47)	(-2.74)	(-2.99)	(-3.32)	(-3.79)
Volume	-0.00326∗∗∗	-0.00324∗∗∗	-0.00331∗∗∗	-0.00340∗∗∗	-0.00385∗∗∗	-0.00361∗∗∗	-0.00347∗∗∗	-0.00350∗∗∗	-0.00357∗∗∗	-0.00397∗∗∗
	(-2.71)	(-2.72)	(-2.78)	(-2.89)	(-3.45)	(-2.95)	(-2.88)	(-2.92)	(-3.01)	(-3.54)
Commissions	-0.00326∗∗∗ (-2.62)	-0.00339∗∗∗ (-2.92)	-0.00349∗∗∗ (-3.10)	-0.00352∗∗∗ (-3.10)	-0.00347∗∗∗ (-2.95)	-0.00341∗∗∗ (-2.71)	-0.00355∗∗∗ (-3.03)	-0.00367∗∗∗ (-3.23)	-0.00374∗∗∗ (-3.26)	-0.00383∗∗∗ (-3.20)

Panel C. 4-Factor Model										
Trades	-0.00300∗∗	-0.00324∗∗∗	-0.00350∗∗∗	-0.00372∗∗∗	-0.00417∗∗∗	-0.00328∗∗	-0.00349∗∗∗	-0.00371∗∗∗	-0.00391∗∗∗	-0.00432∗∗∗
	(-2.37)	(-2.67)	(-2.96)	(-3.31)	(-3.88)	(-2.56)	(-2.84)	(-3.10)	(-3.44)	(-3.98)
Volume	-0.00339∗∗∗	-0.00335∗∗∗	-0.00342∗∗∗	-0.00352∗∗∗	-0.00400∗∗∗	-0.00373∗∗∗	-0.00359∗∗∗	-0.00361∗∗∗	-0.00369∗∗∗	-0.00413∗∗∗
	(-2.82)	(-2.82)	(-2.88)	(-3.00)	(-3.64)	(-3.06)	(-2.98)	(-3.01)	(-3.12)	(-3.73)
Comissions	-0.00341∗∗∗	-0.00352∗∗∗	-0.00361∗∗∗	-0.00363∗∗∗	-0.00360∗∗∗	-0.00355∗∗∗	-0.00367∗∗∗	-0.00379∗∗∗	-0.00386∗∗∗	-0.00396∗∗∗
	(-2.75)	(-3.04)	(-3.21)	(-3.21)	(-3.07)	(-2.84)	(-3.15)	(-3.34)	(-3.38)	(-3.33)

Next, we divide the sample period into two subperiods determined by the bankruptcy of Interbolsa, which occurred in October 2012. Results are shown in Table 5. We find that both alphas were negative, that the alpha was approximately 2% more negative in the first subperiod (01-2006/10-2012) than in the second subperiod (11-2012/01-2016) and that alphas were significant in both subperiods. Additionally, the market betas declined substantially between the first and second subperiods. A Chow structural stability test confirms that both the intercepts and betas are different between the two subperiods (results of the test are not shown, but they are available upon request).

**Table 5:** Individual investors’ performance II. This table reports estimates of the CAPM, the Fama and French 3-factor model and the Carhart 4-factor model for individual investors at the Colombian Stock Exchange. Data are split into two periods: from January 2006 to October 2012, and November 2012 to December 2016, by considering the event of default by the largest stock broker on the Colombian stock market. The dependent variables are the gross and net returns in excess of the one-month Colombian T-bill rate. Market is the return in excess of the Colombian market index (COLCAP) relative to the one-month T-bill rate. SMB is the return on a portfolio of small cap stocks minus big cap stocks. HML is the return on a portfolio long in high book-to-market ratio and short in low book-to-market ratio. WML is the return on a portfolio long on winner stocks during the previous 12 months and short on loser shares during the same period. Note: t-statistics in parentheses. ∗/∗∗/∗∗∗ indicate that the coefficient estimates are significantly different from zero at the 10%, 5%, 1%, respectively.

	Gross risk-adjusted return			Net risk-adjusted return		
	CAPM	3-Factor	4-Factor	CAPM	3-Factor	4-Factor
Panel A. 2006–2012						
Market	0.430∗∗∗	0.449∗∗∗	0.448∗∗∗	0.430∗∗∗	0.448∗∗∗	0.448∗∗∗
	(20.73)	(20.00)	(19.82)	(20.48)	(19.70)	(19.52)
SMB		0.100∗∗ (2.13)	0.0998∗∗ (2.11)		0.0973∗∗ (2.04)	0.0971∗∗ (2.02)
HML		-0.0447	-0.0447		-0.0420	-0.0420
		(-1.15)	(-1.14)		(-1.07)	(-1.06)
WML			-0.00337			-0.00269
			(-0.08)			(-0.07)
Constant	-0.00559∗∗∗	-0.00535∗∗∗	-0.00534∗∗∗	-0.00589∗∗∗	-0.00565∗∗∗	-0.00564∗∗∗
	(-4.27)	(-4.13)	(-4.08)	(-4.45)	(-4.30)	(-4.25)
Observations	81	81	81	81	81	81
R-squared	0.8447	0.8534	0.8534	0.8415	0.8496	0.8496

Panel B. 2012–2016						
Market	0.0773∗∗∗	0.0805∗∗∗	0.0860∗∗∗	0.0773∗∗∗	0.0805∗∗∗	0.0860∗∗∗
	(17.86)	(15.76)	(13.28)	(17.85)	(15.78)	(13.30)
SMB		0.000488	0.00617		0.000544	0.00624
		(0.07)	(0.78)		(0.08)	(0.79)
HML		0.0182∗ (1.80)	0.0190∗ (1.90)		0.0184∗ (1.82)	0.0191∗ (1.91)
WML			0.0100			0.0100
			(1.37)			(1.37)
Constant	-0.00386∗∗∗	-0.00391∗∗∗	-0.00388∗∗∗	-0.00387∗∗∗	-0.00393∗∗∗	-0.00389∗∗∗
	(-18.72)	(-19.03)	(-18.93)	(-18.78)	(-19.12)	(-19.01)
Observations	38	38	38	38	38	38
R-squared	0.8986	0.9102	0.9150	0.8984	0.9103	0.9151

In Table 6, we test for the timing ability of individual investors using the Treynor and Mazuy (1966) and Henriksson and Merton (1981) models for timing for individual investors both on gross and net risk-adjusted returns and for the whole sample period (2006–2016). For both gross and net returns, we find negative and significant coefficients for variable Marketsq (return of the market in excess of the Colombian T-bill rate squared) and negative and significant coefficients for variable Marquet2 (maximum between zero and the negative value of the market). These results suggest that individual investors had bad timing for the whole sample period, resulting in the underperformance of investors. These results explain the nonsignificant coefficients found for the alphas of the four regressions presented in Table 6.

**Table 6:** Timing ability of individual investors. This table reports estimates of the Treynor-Mazuy, and Merton-Henriksson models of timing for individual investors at the Colombian Stock Exchange from 2006 to 2016. The dependent variables are the gross and net returns in excess of the one-month Colombian T-bill rate. Market is the Colombian market index (COLCAP) return in excess of the one-month T-bill rate. Marketsq is the return of the market in excess of the Colombian T-bill rate squared. Market 2 is the maximum between zero and negative values of Market. Note: t-statistics in parentheses. */**/*** indicate that the coefficient estimates are significantly different from zero at the 10%, 5%, 1%, respectively.

		Gross risk-adjusted return	Net risk-adjusted return	
	Treynor Mazuy	Merton Henriksson	Treynor Mazuy	Merton Henriksson
Market	0.329∗∗∗ (15.74)	0.277∗∗∗ (6.61)	0.328∗∗∗ (15.58)	0.276∗∗∗ (6.52)
Marketsq	-0.612∗∗∗ (-3.56)		-0.621∗∗∗ (-3.58)	
Market 2		-0.133∗∗ (-2.16)		-0.135∗∗ (-2.16)
Constant	-0.00174 (-1.38)	-0.000882 (-0.50)	-0.00192 (-1.51)	-0.00105 (-0.59)
Observations	119	119	119	119
R-squared	0.7501	0.7334	0.7468	0.7296

We also ran the same regressions but in this case for the period prior to the bankruptcy of Interbolsa (until October of 2012).^9^ We found that the timing factor still explains the underperformance of investors during the first subperiod (01-2006/10-2012). However, when we run the same regressions for the period following the bankruptcy of Interbolsa (after October of 2012), we find that the sign of the timing factor changes from significantly negative to insignificantly positive in all specifications. This finding is consistent with the possibility that, on average, only the best investors (or the better underperformers) survived the bankruptcy of this important brokerage house in Colombia.^10^

In Table 7, we show the results of running cross-sectional regressions for the whole sample of individual investors (42,211 investors) to analyze whether certain characteristics of investors help explain gross and net returns. Three specifications are run for gross and net returns. In the first specification, we take into account the number of trades performed by each investor, in the second we consider the average number of shares in which each individual invests and, in the third, we consider the relative trading volume of each investor. We find the following results:

1) “Trades” (columns 1 and 4): First, it is confirmed that higher trading intensity (i.e., a higher number of trades) negatively impacts both gross and net returns. Second, investors in the market for longer (variable “Time”) had lower gross returns and higher net returns. This finding suggests that variable Time in the market, which can be regarded as a proxy measure of investor experience, allows the investor to “learn to manage” transaction costs. However, an interaction variable between the number of trades and the length of time an investor was in the market during the sample period (2006–2016) yields a positive and significant coefficient for both the gross and net return regressions. This important finding suggests that investors that are more active in their trading and with more experience in the market are able to earn higher gross and net returns.
2) “Shares” (columns 2 and 5): Variable Shares represents the average number of stocks (companies) that an investor traded during the sample period. This variable can be regarded as a measure of an investor's stock diversification. Regression results indicate that investors who trade in a larger number of stocks tend to have lower gross returns but higher net returns. This evidence suggests that investors “learn to manage” their transaction costs by trading more stocks. An interaction variable between Shares and the lengths of time an investor has been in the market is negative for both specifications, indicating that the performance of investors trading in a greater number of shares and doing so for a longer period was negatively impacted. This result is consistent with other evidence noted earlier (Ivkovic et al., 2008) suggesting that individual investors concentrating their portfolios among a relatively small number of shares tend to outperform other investors, presumably because of the lower information costs that they incur.
3) “Volume” (columns 3 and 6): The results for these regressions are similar to those performed for trades (part 1). This is not surprising, as the correlation between trades and volume is close to 0.9. Once again, the interaction variable (in this case, between variables Volume and Time) suggests that investors that are more active (measured by the volume of stocks traded) and with more experience are able to earn higher gross and net returns.

**Table 7:** Trading impact on individual investors performance. This table reports the impact of trading characteristics on individual investors performance. The dependent variables are the gross and net returns in excess of the one-month Colombian T-bill rate. Trading characteristics are calculated by investor: Trades accounts for the monthly average of transactions. Shares is the number of stocks traded on a monthly basis. Mktsh is the relative traded value per month. Time is the number of months between the first and the last trade. Spread is the average monthly ratio between the daily closing price and the transaction price minus one. For purchases, spread is multiplied by minus one. Note: t-statistics in parentheses. */**/*** indicate that the coefficient estimates are significantly different from zero at the 10%, 5%, 1%, respectively.

	Gross risk-adjusted return			Net risk-adjusted return		
	(1)	(2)	(3)	(4)	(5)	(6)
Trades	-0.000000695∗∗∗ (-7.21)			-0.000000509∗∗∗ (-4.98)		
Trades *×* Time	5.36e-09∗∗∗ (6.29)			3.76e-09∗∗∗ (4.16)		
Shares		-0.00000957∗ (-1.89)			0.0000107∗∗ (1.99)	
Shares *×* Time		-0.000000301∗∗∗ (-5.21)			-0.000000513∗∗∗ (-8.39)	
Mktsh			-1.602∗∗∗ (-4.59)			-0.913∗∗ (-2.47)
Mktsh *×* Time			0.0122∗∗∗ (3.99)			0.00630∗ (1.94)
Time	-0.00000188∗∗∗ (-5.65)	0.00000401∗∗∗ (5.91)	-0.00000172∗∗∗ (-5.23)	0.00000168∗∗∗ (4.75)	0.00000930∗∗∗ (12.96)	0.00000179∗∗∗ (5.14)
Spd	-0.000359 (-0.15)	0.00107 (0.44)	-0.000850 (-0.35)	-0.000842 (-0.33)	0.000671 (0.26)	-0.00129 (-0.50)
Constant	-0.00506∗∗∗ (-200.37)	-0.00513∗∗∗ (-103.72)	-0.00509∗∗∗ (-207.22)	-0.00553∗∗∗ (-206.94)	-0.00576∗∗∗ (-109.99)	-0.00555∗∗∗ (-213.70)
Observations	42,211	42,211	42,211	42,211	42,211	42,211
R-Squared	0.0024	0.0077	0.0015	0.0017	0.0070	0.0011

Finally, and as a last extension, we proceeded to run the regressions of trading intensity (sorted by quintiles) and performance for institutional investors present in the Colombian stock market during the sample period (see Table 8). The results indicate that only institutional investors belonging to the fifth quintile (the quintile corresponding to traders with the most transactions) exhibited alphas that were negative and significant at the 1% level on both a gross and net excess return basis. In the case of the regression for net returns, institutional traders belonging to quintiles 1 and 2 also had negative alphas (on both a 5% and 10% basis, depending on the regression). Overall, the results given in Table 8 suggest that the underperformance of institutional investors in the Colombian stock market (in relation to trading intensity) was less severe than that recorded by individual investors, which is consistent with institutional investors being more informed and better able to handle trading costs than retail investors.

**Table 8:** Institutional investors’ performance by trades. This table reports estimates of the CAPM, the Fama and French 3-factor model and the Carhart 4-factor model for institutional investors at the Colombian Stock Exchange from 2006 to 2016 sorted by quantiles of trades. Panel A exhibits the coefficient estimates on gross excess returns and Panel B on net excess returns. Market is the return in excess of the Colombian market index (COLCAP) relative to the one-month T-bill rate. SMB is the return on a portfolio of small cap stocks minus big cap stocks. HML is the return on a portfolio long in high book-to-market ratio and short in low book-to-market ratio. WML is the return on a portfolio long on winner stocks during the previous 12 months and short on losers shares during the same period. Note: t-statistics in parentheses. ∗/∗∗/∗∗∗ indicate that the coefficient estimates are significantly different from zero at the 10%, 5%, 1%, respectively.

Trades	CAPM		3-Factor				4-Factor				
	Alpha	Market	Alpha	Market	SMB	HML	Alpha	Market	SMB	HML	WML
Panel A. Gross risk-adjusted returns											
Group 1 - Low	-0.00283∗	0.930∗∗∗	-0.00268	0.944∗∗∗	0.0635	-0.0307	-0.00270	0.945∗∗∗	0.0653	-0.0308	0.00838
	(-1.76)	(33.85)	(-1.66)	(30.58)	(1.12)	(-0.57)	(-1.66)	(29.88)	(1.12)	(-0.57)	(0.16)
Group 2	-0.00247	0.948∗∗∗	-0.00258∗	0.940∗∗∗	-0.0438	0.0286	-0.00266∗	0.944∗∗∗	-0.0360	0.0281	0.0371
	(-1.66)	(37.30)	(-1.71)	(32.81)	(-0.83)	(0.57)	(-1.76)	(32.26)	(-0.67)	(0.56)	(0.79)
Group 3	-0.00160	0.984∗∗∗	-0.00166	0.976∗∗∗	-0.0292	-0.00463	-0.00172	0.978∗∗∗	-0.0242	-0.00495	0.0240
	(-1.14)	(41.20)	(-1.18)	(36.23)	(-0.59)	(-0.10)	(-1.21)	(35.51)	(-0.48)	(-0.11)	(0.54)
Group 4	-0.00136	0.915∗∗∗	-0.00149	0.894∗∗∗	-0.0590	-0.0363	-0.00153	0.896∗∗∗	-0.0557	-0.0365	0.0159
	(-1.12)	(44.08)	(-1.24)	(39.02)	(-1.40)	(-0.91)	(-1.26)	(38.19)	(-1.29)	(-0.91)	(0.42)
Group 5 - High	-0.00319∗∗∗	0.698∗∗∗	-0.00329∗∗∗	0.682∗∗∗	-0.0469	-0.0167	-0.00334∗∗∗	0.685∗∗∗	-0.0428	-0.0170	0.0198
	(-3.44)	(44.04)	(-3.57)	(38.84)	(-1.45)	(-0.55)	(-3.60)	(38.11)	(-1.30)	(-0.55)	(0.68)

Panel B. Net risk-adjusted returns											
Group 1 - Low	-0.00348∗∗	0.929∗∗∗	-0.00334∗∗	0.942∗∗∗	0.0594	-0.0287	-0.00335∗∗	0.943∗∗∗	0.0606	-0.0288	0.00596
	(-2.15)	(33.56)	(-2.05)	(30.27)	(1.04)	(-0.53)	(-2.04)	(29.57)	(1.04)	(-0.53)	(0.12)
Group 2	-0.00295∗	0.947∗∗∗	-0.00306∗∗	0.938∗∗∗	-0.0465	0.0325	-0.00315∗∗	0.943∗∗∗	-0.0383	0.0320	0.0390
	(-1.97)	(36.98)	(-2.03)	(32.53)	(-0.88)	(0.65)	(-2.07)	(32.00)	(-0.71)	(0.64)	(0.82)
Group 3	-0.00203	0.985∗∗∗	-0.00210	0.976∗∗∗	-0.0309	-0.00241	-0.00215	0.979∗∗∗	-0.0254	-0.00276	0.0261
	(-1.45)	(41.34)	(-1.49)	(36.34)	(-0.63)	(-0.05)	(-1.52)	(35.64)	(-0.50)	(-0.06)	(0.59)
Group 4	-0.00171	0.915∗∗∗	-0.00184	0.893∗∗∗	-0.0602	-0.0362	-0.00187	0.895∗∗∗	-0.0569	-0.0364	0.0159
	(-1.40)	(44.01)	(-1.53)	(38.97)	(-1.43)	(-0.91)	(-1.55)	(38.14)	(-1.32)	(-0.91)	(0.42)
Group 5 - High	-0.00344∗∗∗	0.698∗∗∗	-0.00355∗∗∗	0.683∗∗∗	-0.0470	-0.0154	-0.00359∗∗∗	0.685∗∗∗	-0.0426	-0.0157	0.0209
	(-3.71)	(44.00)	(-3.84)	(38.79)	(-1.45)	(-0.50)	(-3.88)	(38.08)	(-1.29)	(-0.51)	(0.72)

## 5 Conclusions, possible extensions and implications

In this paper, we have determined that individual investors in the Colombian stock market experienced negative abnormal returns on a gross return basis that ranged between 4% and 4.3% per year (depending on whether the alpha was estimated using the CAPM, the Fama-French model or the Carhart model) between 2006 and 2016. When transaction costs are considered, the underperformance of retail investors becomes even more pronounced, and the most active traders perform worse than less active traders even on a gross excess return basis. These findings are even more disappointing for households when one considers that the COLCAP index declined by 5.8% per year during the period of study. These results were obtained after analyzing the stock transactions executed by the universe of retail investors on the Colombian Stock Exchange during the sample period. The underperformance of households becomes even bleaker when transaction costs are considered.

We found that the average individual investor biases its portfolio toward small, low-beta, and value stocks and turns over approximately 8.8 % of its portfolio monthly. We also determined that the most active traders underperformed relative to less active traders even on a gross return basis (measuring trading activity by either the number of trades or volume) and that bad timing can explain the underperformance of investors, but only prior to the bankruptcy of Interbolsa, which occurred in October of 2012. This finding is consistent with fact that only the best investors (or better underperformers) survived the bankruptcy of this important brokerage house. Importantly, we found that investors that are more active and with more experience in the market were able to earn higher gross and net returns. Finally, we found that institutional investors in the Colombian stock market (in relation to trading intensity) also underperformed but at a less severe level than that recorded by individual investors.

Our results are in conflict with Grossman and Stiglitz's (1980) rational expectations model but can be consistent with models in which investors suffer from overconfidence. It could be argued (but this is only a speculation) that overconfidence could be penalized more severely (in terms of lower gross and net returns) in emerging markets (which tend to be characterized by higher transaction costs, information asymmetries, and corporate governance issues) compared to the case of trading in developed capital markets, where most studies on the performance of individual investors have been undertaken. For the Colombian stock market, Karolyi (2015) finds that while this market fares relatively well in terms of legal investor protection, it still suffers from market capacity constraints, operational inefficiencies and corporate opacity.

Our results are also consistent with evidence presented in a recent paper by Lyngnes (2020), who used unique portfolio holdings data of all domestic retail investors on the Oslo Stock Exchange (OSE) for 1993 to 2006 and found that as investors gain trading experience, their ability to turn portfolio concentration into excess returns improves. The importance of retail investors' experience for performance is also highlighted by the results reported by Arnold et al. (2020), who found that attention triggers (measured using the trading records of a large broker who sends standardized push messages to the cell phones of retail investors) increase investors’ risk-taking and that attention triggers are more relevant to the financial risk-taking of male, less experienced and younger investors. In a similar vein, Barber et al. (2021) find, using data from Robinhood, that investors using this app engage in more attention-induced trading than other retail investors and argue that the evidence is consistent with Robinhood attracting relatively inexperienced investors. As we note above, we found investors with more experience and more active in the market to be able to earn higher gross and net returns. One could argue more experienced investors are able to shield themselves from attention triggers, that this helps them outperform other investors and that this outperformance is augmented as they trade more.

We could extend this study by analyzing whether individual investors that place limit orders underperform relative to informed traders following the study by Linnainmaa (2010) on the Finish Stock Exchange. In the case of the Colombia Stock Exchange, and before February 2009, the market functioned as a continuous trading system from 8:00 to 13:00 on weekdays, and investors were only permitted to place limit orders. However, after February 9, 2009, a new trading platform was introduced, allowing market and stop orders, and a batch auction was introduced during the last five minutes of each trading day (Pedraza et al., 2019). These features of the Colombian stock market would allow us to conduct this study for the period after 2009. According to Wyman (2016), over the last decade, retail investors have been interested in investing in the Colombian Stock Exchange with the launch of a number of “democratization” programs aimed at incorporating new investors into the stock market. For example, in 2007, and as noted above, Ecopetrol, the largest oil company in Colombia, sold stocks through an IPO to almost half a million new investors. A new IPO directed at small investors followed in 2011. While programs such as Ecopetrol were successful in drawing interest from individual investors, they also caused the portfolios of many of these investors to become undiversified. When oil prices declined in late 2014 and the stock prices of oil companies suffered as a result, investors experienced a loss of confidence in the market.

To restore investor confidence in the stock market, the offering of broad financial education programs for small investors will be of upmost importance as well as the reinforcement of regulation and the improvement of corporate governance standards. Regarding the latter issue, Wyman (2016) comments that the Colombian stock exchange still faces a number of challenges that may restrain the tendency to invest in the exchange (most notably, there is a need to reinforce the protection of the rights of minority shareholders so that issuers can draw interest from a wide range of investors), notwithstanding important efforts that have already been made to improve corporate governance practices in Colombia. Furthermore, there is a need to improve liquidity so that transaction costs can be reduced, new investors can be attracted and systemic and liquidity risks can be decreased. The strengthening of the private pension system accompanied by increased interest from foreign investors and new democratization programs will certainly help in increasing liquidity and market participation by retail investors.

## Declarations

### Author contribution statement

Urbi Garay and Fredy Pulga: Conceived and designed the experiments; Performed the experiments; Analyzed and interpreted the data; Contributed reagents, materials, analysis tools or data; Wrote the paper.

### Funding statement

This research did not receive any specific grant from funding agencies in the public, commercial, or not-for-profit sectors.

### Data availability statement

Data will be made available on request.

### Declaration of interests statement

The authors declare no conflict of interest.

### Additional information

No additional information is available for this paper.

## Footnotes

1 We use the terms “retail investors” and “individual investors” interchangeably throughout the text.

2 The following authors also provide literature reviews dedicated to behavioral economics and finance: Rabin (1998), Shiller (1999), Hirshleifer (2001), Daniel et al. (2002), Barberis and Thaler (2003), Campbell (2006), Benartzi and Thaler (2007), and Subrahmanyam (2008).

3 We would like to thank Adriana Cárdenas and other members of the Colombian Stock Exchange Research Committee for kindly providing the data.

4 Short-sales were allowed in the Colombian Stock Exchange during the period of analysis. However, investors had to close short positions by the end of each trading day, and therefore we were not able to use short sale information in our study, as this information is not observable in the database.

5 When the price of a stock changes by more than 10% relative to its reference price, the Colombian Stock Exchange suspends trading in that stock for 30 min. Additionally, all transactions made in the exchange are suspended until the next trading day if the COLCAP index (the local benchmark) declines by more than 10% during a trading session. The COLCAP is a value-weighted index. As of the first quarter of 2018, it included 25 companies, the largest of which was Ecopetrol (12.70% of the total market capitalization of the index), followed by Bancolombia (12.13%).

6 Almost all of these “buy and hold” investors were actually individuals who participated in the IPO of Ecopetrol in 2007 (in which approximately half a million individuals participated, as this IPO was intended to be available to the general public) and did not trade again during the rest of the sample period (or, at most, sold the stocks of Ecopetrol that they had initially bought in the IPO).

7 Domestic institutions (corporations, pension funds, mutual funds, brokerage firms, and others) were responsible for 50% of all transactions (in total value) between 2006 and 2010 and for 27% between 2011 and 2016. Finally, foreign investors accounted for 4% and 17% of all transactions made from 2006-2010 and 2011–2016, respectively. The participation of individual investors among foreign investors was negligible.

8 Overall, coefficients on the factors for each of the three models (the CAPM, Fama and French model, and Carhart model) are similar to those of the main model (Table 3). The results are available upon request.

9 The results are available upon request.

10 We leave for future studies the application of tests using other market timing models.
